# Dosimetry analyses comparing high-dose-rate brachytherapy, administered as monotherapy for localized prostate cancer, with stereotactic body radiation therapy simulated using CyberKnife

**DOI:** 10.1093/jrr/rru048

**Published:** 2014-06-23

**Authors:** Shoichi Fukuda, Yuji Seo, Hiroya Shiomi, Yuji Yamada, Toshiyuki Ogata, Masahiro Morimoto, Koji Konishi, Yasuo Yoshioka, Kazuhiko Ogawa

**Affiliations:** 1 Department of Radiation Oncology, NTT West Osaka Hospital, 2-6-40 Karasugatuji, Tennoji-ku, Osaka 543–8922, Japan; 2 Department of Radiation Oncology, Osaka University Graduate School of Medicine, 2–2 Yamada-oka, Suita, Osaka, Japan; 3 Division of Medical Physics, Oncology Center, Osaka University Hospital, 2–15 Yamada-oka, Suita, Osaka, Japan; 4 Department of Radiology, National Hospital Organization Kinki-chuo Chest Medical Center, 1180 Nakasonecho, Kita-ku, Sakai, Osaka, Japan; 5 Department of Radiation Oncology, Osaka Medical Center for Cancer and Cardiovascular Disease, 1-3-3 Nakamichi, Higashinari-ku, Osaka, Japan

**Keywords:** prostate cancer, stereotactic body radiotherapy, high-dose-rate brachytherapy, hypofractionation, dosimetry

## Abstract

The purpose of this study was to perform dosimetry analyses comparing high-dose-rate brachytherapy (HDR-BT) with simulated stereotactic body radiotherapy (SBRT). We selected six consecutive patients treated with HDR-BT monotherapy in 2010, and a CyberKnife SBRT plan was simulated for each patient using computed tomography images and the contouring set used in the HDR-BT plan for the actual treatment, but adding appropriate planning target volume (PTV) margins for SBRT. Then, dosimetric profiles for PTVs of the rectum, bladder and urethra were compared between the two modalities. The SBRT plan was more homogenous and provided lower dose concentration but better coverage for the PTV. The maximum doses in the rectum were higher in the HDR-BT plans. However, the HDR-BT plan provided a sharper dose fall-off around the PTV, resulting in a significant and considerable difference in volume sparing of the rectum with the appropriate PTV margins added for SBRT. While the rectum D5cm^3^ for HDR-BT and SBRT was 30.7 and 38.3 Gy (*P* < 0.01) and V40 was 16.3 and 20.8 cm^3^ (*P* < 0.01), respectively, SBRT was significantly superior in almost all dosimetric profiles for the bladder and urethra. These results suggest that SBRT as an alternative to HDR-BT in hypofractionated radiotherapy for prostate cancer might have an advantage for bladder and urethra dose sparing, but for the rectum only when proper PTV margins for SBRT are adopted.

## INTRODUCTION

High-dose-rate brachytherapy (HDR-BT) is one of the most effective and precise hypofractionated radiation delivery mechanisms, mainly due to its excellent conformity and rapid falloff outside the target volume. We have treated more than 200 patients with localized prostate cancer using HDR-BT as monotherapy since 1995 and shown that this treatment approach is safe and effective [1]. One disadvantage of HDR-BT, however, is that it requires an invasive procedure under anesthesia to insert the multiple metallic needles. Prolonged bed rest is also necessary following the procedure, resulting in an increased risk of infection or thromboembolism.

Several institutions have utilized stereotactic body radiotherapy (SBRT) for localized prostate cancer [2–5]. The use of this modality is rapidly growing as an alternative approach to HDR-BT. Some trials of SBRT, however, showed higher acute and late toxicity rates when compared with a prolonged course of intensity-modulated radiation therapy (IMRT) or when compared with HDR-BT as monotherapy [6–8]. SBRT and HDR-BT have similar favorable characteristics: higher tumor dose concentration and excellent risk organ dose sparing. SBRT is currently being tested for management of various sites of malignancies, including prostate cancer, using a similar dose regimen to that of HDR-BT. There is also increasing clinical interest in replacing HDR-BT with SBRT boost therapy in the management of other sites of malignancy, including cervical cancer. However, detailed information regarding differences in dosimetric parameters between the two modalities has not been available to date. One study [9] described dosimetric differences between SBRT plans and HDR-BT plans for prostate cancer. The HDR-BT plans were simulated using computed tomography (CT) imaging data of patients treated with SBRT. However, the validity of the study is in doubt, as it adopted the same planning target volume (PTV) margins for SBRT as for HDR-BT, which was found to be inappropriate in a recent study [10]. Further, dosimetric characteristics are highly dependent on needle placement in HDR-BT. In this study, we provide dosimetric analyses comparing HDR-BT with SBRT using common CT images and structure sets for patients treated with HDR-BT; however, in each case the PTV was modified to be valid for SBRT. This is the first report of a dosimetric comparison between real HDR-BT as monotherapy and simulated SBRT for prostate cancer. Since we have long-term clinical experience with hypofractionated treatment via HDR-BT, the data from this study may provide useful information for the design of SBRT for localized prostate cancer.

## MATERIALS AND METHODS

We selected six consecutive patients with localized prostate cancer treated by HDR-BT from January through June 2010 with informed consent. All patients had biopsy-proven adenocarcinoma of the prostate. According to the 2002 International Union Against Cancer TNM staging system, four patients had Stage T2a, and two patients had T3a. We deﬁned intermediate-risk patients as those with a pre-treatment prostatic serum antigen (iPSA) level of ≥10 but <20 ng/ml, Gleason Score 7, or Stage T2b–T2c disease; high-risk patients were defined as those with an iPSA level of ≥20.0 ng/ml, Gleason Score ≥ 8, or Stage T3–T4 disease. Of the six patients, two were classiﬁed as intermediate risk and four were classified as high risk.

### Implant technique

We have previously described the implant technique in detail [1]. In brief, this technique involved continuous epidural anesthesia, real-time transrectal ultrasound (TRUS) guidance, and the use of metallic applicators. Under real-time TRUS monitoring of the largest cross-section of the prostate, the applicators were placed on the line encompassing the prostate (with or without extracapsular invasion) as well as inside the prostate but sparing the urethra, at 1- to 1.5-cm intervals. For the posterior (rectal) side, the applicators were placed 0–3 mm inside the prostatic capsule. The top 2 cm of the catheters penetrated through the prostate gland and were placed within the bladder pouch.

### HDR-BT treatment planning

**Fig. 1.**
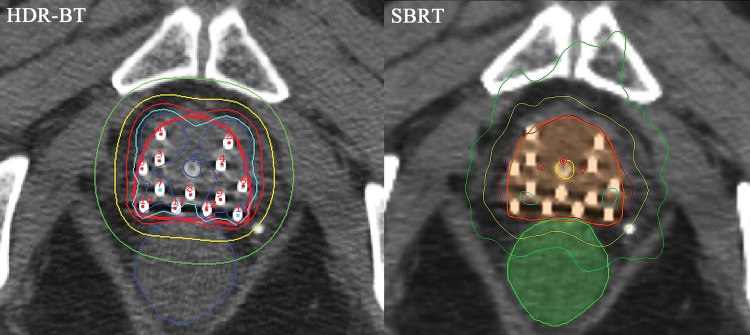
Axial comparison: HDR-BT vs simulated SBRT. Bold line (red for HDR-BT; orange for SBRT) = PTV. Isodose lines shown as follows: 150% = dark blue; 125% = light blue; 100% = red; 75% = yellow; 50% = green. Note HDR-BT 125% and 100% isodose lines at the ventral portion of the prostate are horseshoe-shaped because of insufficient needle insertion.

The clinical target volume (CTV) comprised the whole prostate gland plus 5 mm in all directions to cover possible extracapsular extensions [11], except for the posterior (rectal) margin. The posterior margin varied from 2 to 5 mm depending on the distance from the rectal wall. If extracapsular invasion was observed or strongly suspected, the area with the margin was included in the CTV. The PTV for HDR-BT was equal to the CTV except in the cranial direction, where it was expanded 5 to 10 mm from the CTV. This margin was established to prevent creation of a cold area at the base of the prostate and also for insurance against the possibility of catheter displacement during the treatment course. In Fuller's study [9], the PTV for SBRT equaled that of HDR-BT; however, we adopted a different PTV margin for SBRT, because SBRT needs extra PTV margins due to intrafraction motion of the prostate. Xie *et al*. reported that orthogonal imaging every 30–60 s using the CyberKnife (CK) treatment system would allow 95.6% and 92.5% of the beams to be delivered within 2 mm of the target during CK treatments of the prostate. Therefore, we adopted 2-mm PTV margins in all directions from the CTV for SBRT. The urethra was delineated from the membranous portion to the bladder neck by identifying the Foley catheter. The rectum was delineated as a solid organ (not as the rectal wall) from just above the anal verge to 3 cm cranial to the PTV on the CT images, so it may have included a caudal part of the sigmoid colon. This was because we needed to evaluate the radiation dose deposit spreading out in both cranial and caudal directions in SBRT plans that utilize non-coplanar external beams. The bladder was delineated in all slices of the CT images as a solid organ. Treatment planning was done with the aid of a computer-assisted planning system (PLATO; Nucletron) using geometric optimization. The dose prescription point was at 5 mm lateral from one peripheral source on the central plane. The source dwell positions were located on the surface and inside the prostate. The prescription dose was 45.5 Gy in seven fractions. We attempted to achieve a PTV V100 (percentage of volume of interest receiving 100% of the prescribed radiation dose) of 100%. However, it was difficult to accomplish this in some cases, because the urethra, running anterior at the spongy part, interfered with metallic needle placement in the medial ventral part of the prostate (Fig. 1). In such cases, we accepted a partially lower dose coverage. The dose to the whole urethra should be between 100 and 150% of the prescription dose, and the dose to the whole rectal area should be <100% of the prescription dose.

### Dosimetry comparison

A simulated SBRT plan for each HDR-BT plan was designed on the CK treatment-planning system (MultiPlan, Accuray Inc., Japan) with the common CT images and contouring sets (except for the PTV) of the corresponding HDR-BT plan used for the actual treatment. The dose constraints on SBRT planning were as follows: the PTV dose was constrained to be between 100 and 150% of the prescription dose. It was also required that the maximum rectal, urethra and bladder doses were each ≤100% of the prescription dose. In addition, in order to prevent sexual dysfunction, no beams exited through the testes, according to a cautionary note [12]. The bilateral femoral heads dose was required to be <28 Gy. For each of the delivered HDR-BT plans, the radiation dose covering 95% of the PTV (D95%) was calculated. The D95% was used as the prescription dose for each SBRT plan to compare dosimetry of the PTV and organ at risk for HDR-BT with those of SBRT. This is because the D95% is one of the most common dose prescription methods in recent radiation therapy and because it is hard for the biological effect on the prostate to be made equivalent for HDR-BT and SBRT due to the fundamental difference in physical characteristics between brachytherapy and external beam therapy.

Dosimetric values for SBRT and HDR-BT plans were evaluated and compared with respect to PTV coverage (V100, V125, V150, D90%, D95%, D99%, D100%), rectal exposure (volume [cm^3^] of interest receiving 20% of prescribed radiation dose [V20], V40, V50, V60, V80, V100, minimal radiation dose for the most irradiated rectal volumes of 0.1 cm^3^ [D0.1cm^3^], D1cm^3^, D2cm^3^, D5cm^3^, D10cm^3^, D20cm^3^, D30cm^3^, D40cm^3^, D50cm^3^), urethra exposure (D0.1cm^3^, D0.2cm^3^, D0.5cm^3^, D10%, D20%), and bladder exposure (V20, V40, V60, V80, V100, D0.5cm^3^, D1cm^3^, D2cm^3^, D5cm^3^, D10cm^3^, D20cm^3^, D40cm^3^, D80cm^3^).

All statistical analyses were done by paired *t*-test.

## RESULTS

### PTV coverage

**Fig. 2.**
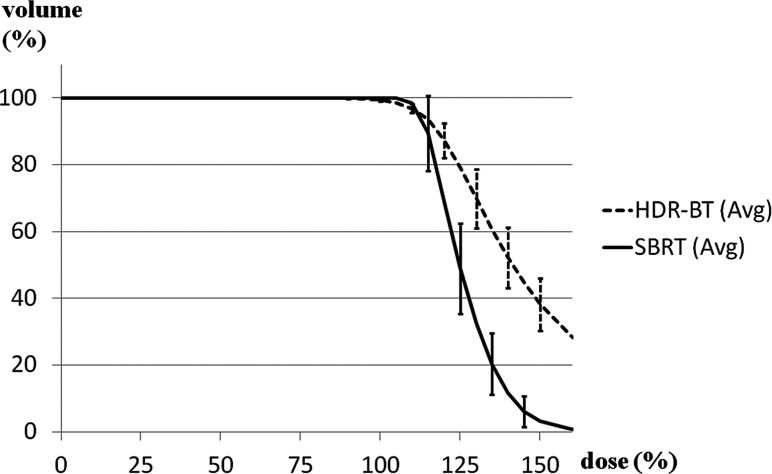
Comparison of dose–volume histograms of the PTV for SBRT (solid line) and HDR-BT (dashed line). HDR-BT had superior dose concentration and worse homogeneity and prescription dose coverage. SBRT (Avg) = average value of SBRT, HDR-BT (Avg) = average value of HDR-BT.

HDR-BT had superior dose concentration but worse homogeneity and prescription dose coverage. The average prescription dose for the HDR-BT plans was 51.8 Gy (49.9–53.3 Gy). The PTV coverage comparisons are shown in Table 1 and Fig. 2. The average V100 values of the actual HDR-BT and simulated SBRT plans measured 99.5 and 100%, respectively. Although there was a statistically significant difference (*P* < 0.01) with paired observations, the difference in V100 values was quantitatively small. Thus, one may consider that both plans covered the targets quite well. The average V125 and V150 values were significantly higher in HDR-BT (79.4 vs 48.9% for HDR-BT and SBRT, respectively, *P* < 0.01; and 40.8 vs 3.1%, respectively, *P* < 0.01). Moreover, the average D90% was significantly higher in HDR-BT (54.1 vs 52.4 Gy, *P* < 0.01), but the average D99% and D100% were less for HDR-BT relative to SBRT by 3.9 Gy and 8.9 Gy, respectively. These data indicated greater dose heterogeneity inside the PTV for HDR-BT than for SBRT. Isodose lines on the CT-based treatment plans of the two modalities are illustrated at 150% (dark blue), 125% (light blue), 100% (red), 75% (yellow), and 50% (green) (Fig. 1).

**Table 1.** Summary of dose–volume parameters for simulated CK SBRT and HDR-BT for the PTV

Structure	Parameter	Unit	HDR-BT	CK SBRT	*P*
**PTV**					
	D90%	Gy	54.1 ± 1.6	52.4 ± 1.3	<0.01
	D95%	Gy	51.8 ± 1.3	51.8 ± 1.3	
	D99%	Gy	47.1 ± 1.2	51.0 ± 1.3	<0.01
	D100%	Gy	40.0 ± 3.0	48.9 ± 1.7	<0.01
	V100	%	99.5 ± 0.3	100.0 ± 0.0	<0.01
	V125	%	79.4 ± 7.2	48.9 ± 13.6	<0.01
	V150	%	40.8 ± 7.9	3.1 ± 3.1	<0.01
	V200	%	15.3 ± 3.3	0 ± 0	<0.01

HDR-BT = high-dose-rate brachytherapy, CK SBRT = CyberKnife stereotactic body radiotherapy, PTV = planning target volume, D*x*% = the radiation dose covering *x*% of the PTV, V*x*% = percentage of normalized volume of interest receiving *x*% of prescribed radiation dose. Prescription dose was 45.5 Gy in seven fractions.

### Rectum

**Fig. 3.**
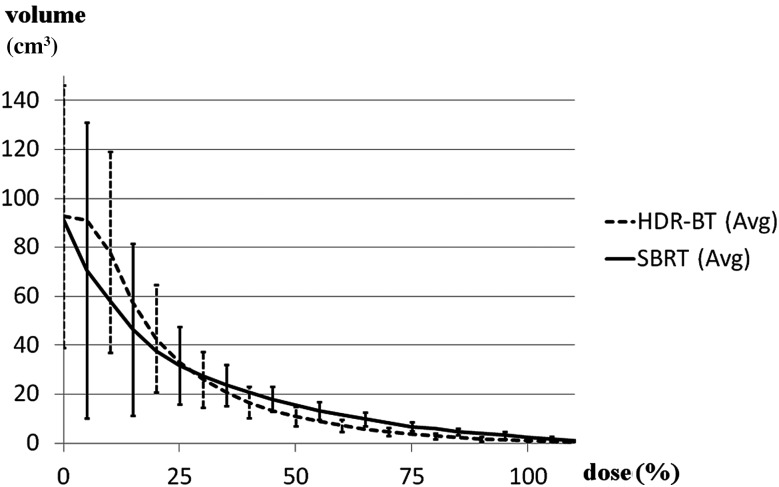
Comparison of dose–volume histograms of the rectum for SBRT (solid line) and HDR-BT (dashed line). HDR-BT had steeper fall-off inside the rectum than SBRT in the intermediate to high dose range. SBRT (Avg) = average value of SBRT; HDR-BT (Avg) = average value of HDR-BT.

HDR-BT had a higher maximum dose and a steeper fall-off inside the rectum than SBRT in the intermediate to high dose range. The rectum dosimetry values are shown in Table 2 and Fig. 3. The average D0.1cm^3^ values (nearly maximum dose of interest) were higher in HDR-BT (58.3 vs 52.3 Gy, respectively, *P* = 0.21). However, as shown in Table 2, both lines intersected between D0.1cm^3^ and D1cm^3^. The average D1cm^3^, D2cm^3^, D5cm^3^, D10cm^3^ and D20cm^3^ values were significantly lower in HDR-BT by 5.1, 7.1, 7.6, 6.1 and 2.4 Gy, respectively. The average V40–100 values were significantly lower in HDR-BT, although the differences were relatively small (1.6–4.5 cm^3^).

**Table 2.** Summary of dose–volume parameters for simulated CK SBRT and HDR-BT for organs at risk

Structure	Parameter	Unit	HDR-BT	CK SBRT	*P*
**Rectum**					
	D0.1cm^3^	(Gy)	58.3 ± 9.9	52.3 ± 1.3	0.21
	D1cm^3^		44.1 ± 3.8	49.2 ± 2.7	0.01
	D2cm^3^		39.1 ± 3.9	46.2 ± 3.5	<0.01
	D5cm^3^		30.7 ± 3.8	38.3 ± 4.0	<0.01
	D10cm^3^		23.0 ± 3.4	29.1 ± 3.6	<0.01
	D20cm^3^		15.4 ± 3.1	17.8 ± 4.1	0.01
	D30cm^3^		11.2 ± 3.3	10.0 ± 5.1	0.29
	D40cm^3^		8.6 ± 3.3	5.8 ± 5.2	0.04
	D50cm^3^		6.9 ± 3.3	3.8 ± 5.0	0.02
	V20	(cm^3^)	41.7 ± 21.5	37.8 ± 23.7	0.18
	V40		16.3 ± 6.2	20.8 ± 6.5	<0.01
	V50		10.6 ± 3.7	15.6 ± 4.3	<0.01
	V60		7.1 ± 2.5	11.5 ± 3.1	<0.01
	V80		2.9 ± 1.3	6.0 ± 2.0	<0.01
	V100		0.9 ± 0.7	2.5 ± 1.1	<0.01
**Urethra**					
	D0.1cm^3^	(Gy)	65.2 ± 6.9	52.6 ± 1.2	<0.01
	D0.2cm^3^		61.3 ± 4.2	52.4 ± 1.2	<0.01
	D0.5cm^3^		56.3 ± 2.8	51.8 ± 1.2	<0.01
	D10%		62.7 ± 4.6	52.5 ± 1.2	<0.01
	D20%		59.1 ± 3.5	52.1 ± 1.2	<0.01
**Bladder**					
	D0.5cm^3^	(Gy)	96.2 ± 36.8	51.1 ± 6.1	0.02
	D1cm^3^		76.4 ± 25.4	48.6 ± 6.8	0.03
	D2cm^3^		61.2 ± 16.6	45.0 ± 7.4	0.03
	D5cm^3^		45.8 ± 10.5	38.4 ± 6.7	0.08
	D10cm^3^		35.0 ± 7.4	31.3 ± 5.2	0.18
	D20cm^3^		25.2 ± 5.2	24.1 ± 3.9	0.57
	D40cm^3^		17.0 ± 2.9	16.7 ± 3.4	0.84
	D80cm^3^		10.5 ± 1.2	6.2 ± 2.3	0.01
	V20	(cm^3^)	94.2 ± 13.3	66.1 ± 12.8	0.02
	V40		35.2 ± 10.0	35.8 ± 10.8	0.91
	V60		17.0 ± 6.9	14.8 ± 6.8	0.39
	V80		9.3 ± 4.9	6.6 ± 3.5	0.12
	V100		5.2 ± 3.4	2.6 ± 1.7	0.07

D*x*cm^3^ = minimal radiation dose for the most irradiated organ volumes of *x* cm^3^, Vx = volume (cm^3^) of interest receiving *x*% of prescribed radiation dose, D*x*% = the radiation dose covering *x*% of the urethra. Prescription dose was 45.5 Gy in seven fractions.

### Urethra

**Fig. 4.**
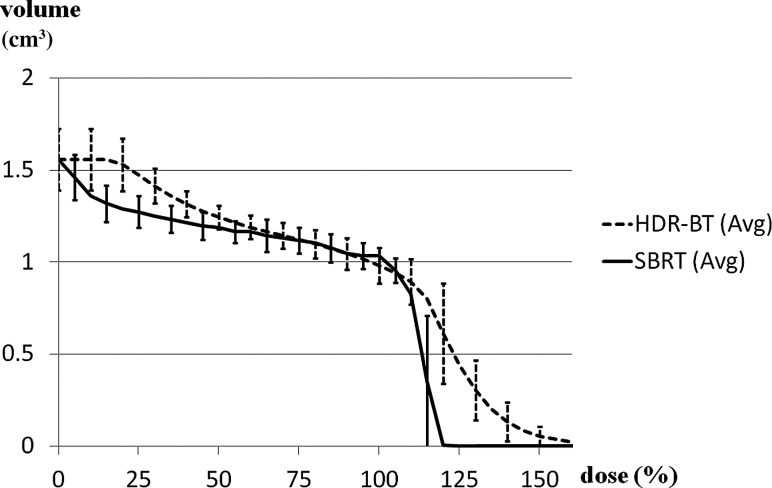
Comparison of dose–volume histograms of the urethra for SBRT (solid line) and HDR-BT (dashed line). HDR-BT was far inferior to SBRT at high dose levels. SBRT (Avg) = average dose of SBRT; HDR-BT (Avg) = average dose of HDR-BT.

HDR-BT was inferior to SBRT for all dosimetric parameters for the urethra. The urethra dosimetry values are shown in Table 2 and Fig. 4. The average urethra D0.1cm^3^, D0.2cm^3^, D0.5cm^3^, D10% and D20% were considerably higher in HDR-BT than in SBRT by 12.6, 8.9, 4.5, 10.2 and 7.0 Gy, respectively. The differences for all urethra dosimetry parameters were statistically significant.

### Bladder

**Fig. 5.**
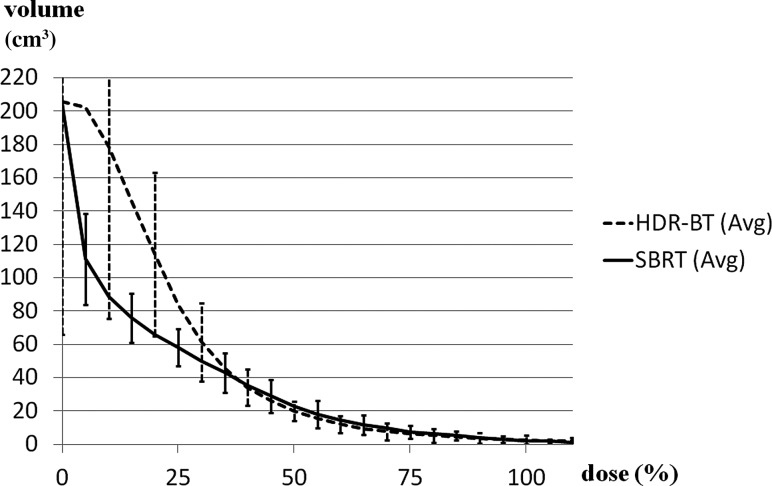
Comparison of dose–volume histograms of the bladder for SBRT (solid line) and HDR-BT (dashed line). HDR-BT had a much higher maximum dose and steeper fall-off. However, the inferiority of HDR-BT to SBRT was maintained at a lower dose range. SBRT (Avg) = average value of SBRT; HDR-BT (Avg) = average value of HDR-BT.

HDR-BT was also inferior to SBRT for all dosimetric parameters for the bladder. Bladder dosimetry values are shown in Table 2 and Fig. 5. HDR-BT had a much higher maximum dose. The average D0.5cm^3^ values (assumed to be nearly the maximum doses of interest) were 96.2 Gy and 51.1 Gy for HDR-BT and SBRT, respectively (*P* = 0.02). HDR-BT showed a steeper fall-off, but the inferiority of HDR-BT to SBRT was maintained in the lower dose range.

## DISCUSSION

### Difference in PTV dose distribution and its clinical significance

Both the HDR-BT and simulated SBRT plans showed adequate dose coverage for the PTV, although the simulated SBRT plan was numerically slightly better with respect to PTV coverage than the HDR-BT plan. In our HDR-BT plan, a small cold spot was present in the medial ventral part of the PTV, where metallic needles were difficult to place (Fig. 1). The clinical importance of covering that area, which is separate from the peripheral zone, is limited except for cases with visible invasion. Since dose escalation to the prostate leads to better outcome [13, 14], much higher V125 and V150 values in HDR-BT could be a potential advantage when compared with SBRT. However, the isoeffective dose of 51.8 Gy in seven fractions is ∼132 Gy if given at 2 Gy per fraction, according to the linear–quadratic model with an a/b ratio of 1.5 Gy, or 108 Gy with an β ratio of 3 Gy. That would be a much higher biologically effective dose than the commonly prescribed dose of 74–80 Gy using IMRT. Therefore, it is unclear whether further attempts to increase the V125 and V150 values in SBRT to the levels of those in HDR-BT are of clinical benefit.

### Dose difference in normal tissues

We previously reported that D1cm^3^–D10cm^3^ were significantly associated with rectal bleeding, while D5%–D90% were not [15]. Therefore, we suggested D5cm^3^ and V40 for the rectum constraints. Similarly, the European Group of Curietherapie–European Society for Therapeutic Radiology and Oncology (GEC–ESTRO) working group for gynecological brachytherapy recommended D0.1cm^3^, D1cm^3^, D2cm^3^, D5cm^3^ and D10cm^3^ to organs at risk, rather than D5% and D10% [16]. Akimoto [17] also demonstrated V10, V30 and V50 as predictive dosimetric factors for rectum toxicities. This illustrates the relative importance of D*x*cm^3^ or V*x*% when compared with D*x*% that would be highly dependent on an absolute volume of the rectum. In this study, D1cm^3^–D20cm^3^, D40cm^3^, D50cm^3^ and V40–100 values in SBRT were significantly greater than those in HDR-BT with appropriate PTV margins for SBRT (Table 2). This result indicates a statistically better rectal sparing for HDR-BT than for SBRT. Since the differences between the two modalities appeared to be considerable, one may consider that SBRT cannot provide comparable dose sparing for the rectum under the condition of appropriate PTV margins being added for SBRT.

For urinary toxicities, urethra D10 and D20 values were reported to be significantly higher in the patients with Grade 2–3 acute toxicity [18]. In our study, SBRT was superior to HDR-BT in all urethra and bladder parameters. This is mainly because the 5–10 mm PTV margin of HDR-BT in the cranial direction, which is intrinsically peculiar to HDR-BT, might have a negative impact on HDR-BT dosimetry with respect to sparing the bladder.

One limitation of this study is the arbitrary manner in which dosimetry was compared between HDR-BT and SBRT. We adopted the D95% value from each HDR-BT plan for the prescription dose of each SBRT plan because the biological effects to the prostate for HDR-BT and SBRT were hard to make equivalent to each other, due to the fundamental difference in physical characteristics between brachytherapy and external beam therapy.

Another limitation of the present study was the use of manual optimization in HDR planning. Some studies have reported that inverse optimization might be superior to manual optimization for dose sparing of organs at risk without sacrificing PTV dose conformity [19, 20]. At present, however, inverse planning is not available in our institution due to time restrictions in real implants. Likewise, it is also possible that more effective SBRT inverse planning and radiation beam set could result in superior radiation dosimetry.

Another limitation is that this study did not take into account targeting inaccuracy and dosimetry errors in SBRT and HDR-BT. Each modality has different kinds of targeting inaccuracy and dosimetry errors; for example, HDR-BT is subject to longitudinal HDR catheter displacement, prostate volumetric change caused by prostate compression by needle insertion, and prostate edema after irradiation during the treatment course [21], while SBRT is subject to unpredictable intrafraction motion error under rapid and complicated prostate movement caused by bowel peristalsis, passing of gas and bladder filling. Since the sources of dosimetry errors and targeting inaccuracy differ between these two modalities, it is not known which modality can deliver its treatment plan more accurately.

Published late rectal and urinary toxicity rates following SBRT and HDR-BT are summarized in Table 3. Madsen *et al*. [2] reported a relatively high late Grade 2 rectal toxicity rate of 7.5% when compared with 2.0–2.5% in other SBRT trials reported by King *et al*. [5] and Freeman *et al*. [4]. However, the former prescription dose was 33.5 Gy in five fractions, which is lower than that used in the latter prescription dose (35–36.25 Gy in five fractions). Another major difference in their SBRT was the linac systems; Madsen used a conventional linear accelerator system and SBRT plans involving six non-coplanar beams, while King and Freeman used the CK system. In Madsen's report, the average rectum V100, V80 and V50 values were 1.06 ± 1.18, 5.20 ± 3.93 and 10.64 ± 6.83 cm^3^, respectively, whereas our simulated CK SBRT data showed values of 2.2 ± 1.0, 5.6 ± 2.0 and 15.1 ± 4.1 cm^3^, respectively. Madsen's SBRT showed larger standard deviations relative to the average values and a larger number of patients when compared with those for SBRT in our study. SBRT by CK are delivered by more than 100 beams from virtually unlimited directions and has excellent dose conformity. For this reason, dose distributions of CK SBRT are less subject to anatomic variations in the target and the organs at risk of patients. Madsen's SBRT was delivered by only six non-coplanar beams, which might have caused insufficient organ-at-risk dose sparing in some patients. It would be a matter for speculation that this might account for why late rectal toxicities for Madsen were relatively high. In terms of HDR-BT, late rectal toxicity rates were 0–7.0% [6–8], which are comparable to the external beam radiation therapies [2, 4, 5].

**Table 3.** Late rectal and urinary toxicity rates of SBRT and HDR-BT

Institute Author (year), prescription dose	GU grade (%)			GI grade (%)			MFT (months)
	I	II	III	I	II	III	
**SBRT**							
Madsen (2007) [2], 33.5 Gy/5 Fr, *n* = 40	25.0	20.0	0.0	30.0	7.5	0.0	41
Freeman (2011) [4], 35–36.25 Gy/5 Fr, *n* = 41	25.0	7.0	2.5	13.0	2.5	0.0	60
King (2012) [5], 36.25 Gy/5 Fr, *n* = 67	23.0	5.0	3.5	14.0	2.0	0.0	32
**HDR-BT**							
Grills (2004) [6], 38 Gy/4 Fr, *n* = 65	52.0	21.0	8.0	15.0	0.0	0.0	35
Ghdjar (2009) [7], 38 Gy/4 Fr, *n* = 36	36.1	11.1	5.6	0.0	5.6	0.0	36
Yoshioka (2010) [8], 54 Gy/9 Fr, *n* = 112	N/A	6.0	1.0	N/A	5.0	2.0	65

SBRT = stereotactic body radiotherapy, HDR-BT = high-dose-rate brachytherapy, GU = genitourinary toxicity, GI = gastrointestinal toxicity, MFT = median follow-up time, N/A = not available.

We also reported a low rate of urinary late toxicities (6%) by HDR-BT when given at 54 Gy in nine fractions [1], and toxicity rates in an ongoing protocol delivering 45.5 Gy in seven fractions are even lower (data not shown). However, in recent reports of HDR-BT as monotherapy by Grills *et al*. [6] and Ghadjar *et al*. [7], the rate of Grade 2 late urinary toxicities is 11–21%, which is higher than those associated with SBRT (5–7%). This high incidence of late urinary toxicities might have resulted from a technical variation, but may also be consistent with the superiority of SBRT over HDR-BT in terms of urethra dose sparing.

In conclusion, we demonstrated differences in the target and organ-at-risk dose distribution between HDR-BT and simulated SBRT with common CT images and structure sets for patients who received HDR-BT with appropriate PTV margins for SBRT. There were statistically significant differences in the dosimetric parameters inside as well as outside the PTV when a similar marginal dose was delivered. The clinical relevance of these differences has not yet been well defined, and the available clinical outcome data at present appear to indicate that we should pay careful attention when we design SBRT in hypofractionated radiotherapy for prostate cancer.

## FUNDING

This work was in part supported by Osaka University.
